# Genetic Insights into Bicuspid Aortic Valve Formation

**DOI:** 10.1155/2012/180297

**Published:** 2012-06-04

**Authors:** Brigitte Laforest, Mona Nemer

**Affiliations:** Department of Biochemistry, Microbiology and Immunology, University of Ottawa, Ottawa, ON, Canada K1N 6N5

## Abstract

Bicuspid aortic valve (BAV) is the most common congenital heart defect, affecting 1-2% of the population. It is generally diagnosed late in adulthood when deterioration of the abnormal leaflet becomes clinically evident. BAV patients have an increased risk of developing serious complications, including stenosis, regurgitation, endocarditis, dilation of the aorta, aortic dissection, and aneurysm. BAV is a heritable trait, but the genetic basis underlying this cardiac malformation remains poorly understood. In the last decade, thanks to studies in animal models as well as genetic and biochemical approaches, a large number of genes that play important roles in heart development have been identified. These discoveries provided valuable insight into cardiac morphogenesis and uncovered congenital-heart-disease-causing genes. This paper will summarize the current knowledge of valve morphogenesis as well as our current understanding of the genetic pathways involved in BAV formation. The impact of these advances on human health including diagnosis of BAV and prevention of cardiovascular complications in individuals with BAV or with a family history of BAV is also discussed.

## 1. Overview of Valve Development

In human and other mammals, cardiac valves are essential for unidirectional blood flow which is crucial for proper functioning and survival of the organism. Defects in valve structure or function can profoundly alter cardiovascular homeostasis and are among the leading causes of human morbidity and mortality. Thus, understanding the molecular basis of normal and pathologic valve development is of utmost importance and clinical relevance. The valves of the four chambered heart can be classified in 2 groups: atrioventricular valves (mitral and tricuspid) separate the atria from the ventricles while semilunar valves (aortic and pulmonary) divide the ventricles from the great arteries. Normal aortic valve is tricuspid, meaning it possesses three leaflets (or cusps) but defective development involving fusion of two of these produces bicuspid aortic valve (BAV). At 1-2% prevalence in the human population, BAV is the most common form of congenital heart disease and a significant risk factor for premature cardiovascular complications and valve replacement in young adults.

In the embryo, the heart is the first organ to develop starting with the specification and migration of the anterior lateral plate mesoderm cells to form the cardiac crescent [1]. This is closely followed (embryonic day (E) 8.5 in the mouse and 3 weeks gestation in human) by migration of the cardiac progenitors along the ventral midline where they fuse to form a beating linear heart tube. This primitive tube is composed of an inner endocardial cell layer separated by the extracellular matrix (ECM), also known as the cardiac jelly from the outer myocardial lining. At around E9.5 in the mouse and 4-5 weeks in human, cardiac looping occurs and brings the atrial region of the linear tube into a posterior position to the common ventricle. At the same time, increased production of ECM will cause the tissue to swell at localized regions of the heart, initiating the formation of the endocardial cushions at the atrioventricular (AV) canal and outflow tract (OFT) [2, 3]. Endocardial cushion formation is initiated by signals emanating from the AV canal and OFT myocardium that will diffuse into the ECM and reach the adjacent endocardial cells to undergo EMT. A large number of transcription factors and signaling pathways have been implicated in EMT and cushion morphogenesis, including members of the TGF-*β* superfamily, VEGF, ErbB, NFATc1, Notch, Wnt/*β*-catenin, Twist-1, Sox9, Tbx20, and Gata4, and their function has been nicely described in a recent review [4].

Endocardial cushion formation is a complex process that relies on the transformation of a subpopulation of inner endocardial cells into mesenchymal cells; this process termed epithelial-to-mesenchymal transformation (EMT) occurs at the AV canal boundary to initiate mitral and tricuspid valve formation and, later, in the OFT to initiate aortic and pulmonary valve formation. As development progresses, these cushions will undergo exhaustive cell proliferation and grow towards each other until they touch initiating a process of fusion between the two cushions. Further remodeling of the endocardial cushions results in the formation of thin protruding leaflets composed of endocardial cells and ECM that go on to form the valves. These last steps are dependent on cell differentiation, apoptosis and ECM remodeling. In the AV canal, EMT-derived mesenchymal cells are the sole contributor to the mitral and tricuspid valves. In the OFT, mesenchymal cells originating from migrating cardiac neural crest cells will reach the OFT cushions and together with endocardially derived mesenchymal cells will contribute to the formation of the aortic and pulmonary valves. It is of no surprise then that subtle perturbations in endocardial cushion development can lead to heart valve diseases, including bicuspid aortic valve (BAV). Of note, valve defects are among the most frequent cardiovascular malformations accounting for 25–30% [5].

**Figure 1:**
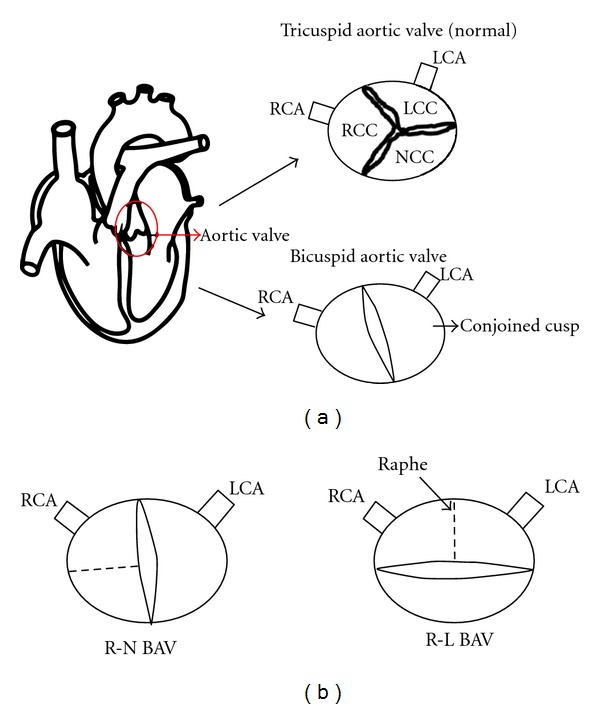
Schematic representation of a bicuspid aortic valve. (a) Transverse section through the aorta showing a normal aortic valve with three leaflets and their corresponding points of attachment to the aortic wall. A bicuspid aortic valve arises from fusion of two different cusps, resulting in the formation of a conjoined leaflet. (b) Schematic representation of the BAV subtypes. The R-N BAV arises from fusion of the right coronary cusp and noncoronary cusp whereas the R-L BAV results from the fusion of the right coronary cusp and left coronary cusp. LCA: left coronary artery; LCC: left coronary cusp; NCC: noncoronary cusp; RCA: right coronary artery; RCC: right coronary cusp.

In normal individuals, the aortic valve possesses three leaflets, namely, the left coronary, the right coronary, and noncoronary cusps, named after their relationship to the coronary arteries (Figure 1). The morphology of the BAV usually includes leaflets of unequal size as the result of the fusion of two cusps, leaving one leaflet larger than the other [6]. In humans, the larger leaflet is characterized by a raphe, which is a thin ridge of tissue that represents the location where the two cusps fused during valve development. In addition, leaflet orientation has been shown to vary widely among patients. The most frequent BAV subtypes are those with fusion of the right and left (R-L) coronary leaflet, representing 59% of cases and those with union of the right and noncoronary (R-N) leaflet occurring in 37% of BAV cases [7]. Insight into the etiology of the R-N and R-L BAVs was further gained from studies of *Nos3* null mice as well as the inbred Syrian hamsters strain which selectively display R-N and R-L subtypes, respectively [8, 9]. These studies suggested that the R-N BAV is caused by defective formation of the OFT cushion whereas the R-L BAV is likely the result of defective OFT septation. Thus, the two BAV subtypes appear to have distinct etiologies. In addition, it has been shown that the R-N fusion is associated with increased dimensions of the aortic root as well as development of aortic insufficiency later in life [10, 11]. The R-L fusion, on the other hand, is associated with a more rapid progression to valve dysfunction and increased dimension of the aortic arch. A new technique, called 3D time resolved phase contrast cardiac magnetic resonance (4D Flow), has been described recently as a powerful tool to allow a better characterization of aortic-valve-related flow dynamics as well as progression of aortic dilation in patients with BAVs [11]. In this study, Hope et al. demonstrated a significant elevation of ascending aortic wall shear stress in a subgroup of patients with BAVs, the majority of whom had R-L fusion. Shear stress to the aortic wall has been linked to aortic aneurysm formation, thus this technique provides a noninvasive mean to quantify the increased blood flow through the ascending aorta in patients with BAVs even before there is a clear manifestation of aortic aneurysms.

## 2. Insights from Human Genetics

Information regarding prevalence of human BAVs has been acquired through autopsy and echocardiographic studies. BAV is now believed to be the most common cardiovascular malformation with a prevalence of 1-2% in the general population [12]. Even though BAV is usually an isolated defect, it may be associated with other cardiovascular syndromes or malformations with at least one-third of patients likely to develop serious complications that will require valve surgery [13]. It is now clear that BAV is a risk factor for adult cardiovascular events. These include aortic stenosis, regurgitation, dissection, dilation/aneurysm as well as valve calcification and infective endocarditis. In the last decade, conflicting results have been obtained regarding the presence of thoracic aortic aneurysms in the setting of BAVs [14]. Some groups have shown that there is a higher rate of aortic root dilatation in individuals with BAVs while others found thoracic aortic aneurysms in first-degree relatives with normal tricuspid aortic valves. Thus, aortic aneurysms in the context of a BAV may not exclusively be due to disturbed blood flow through the aortic valve. For example, Bunton et al. have demonstrated that the fibrilin-1 gene, which is involved in Marfan syndrome, plays an important role in maintaining the integrity of elastic fibers and that mutations in *FIBRILIN-1* could decrease the elastin content in the aorta, providing a new mechanism for the presence of aneurysms in patients with Marfan syndrome [15]. A better understanding of the molecular pathways that maintain the integrity of the aorta is warranted in order to elucidate the pathogenesis of aortic aneurysms in BAV patients.

Several studies addressing the heritability of BAV revealed high incidence of familial clustering [16–18]. Glick and Roberts noted that 17 patients out of 71 family members (24%) had aortic valve disease likely secondary to a BAV, with 2 or more family members affected. Using echocardiography screening of 190 first-degree relatives, Huntington and colleagues reported a prevalence of BAV of 9.1% among the first-degree relatives, suggesting that the distribution of BAV follows an autosomal dominant inheritance with reduced penetrance [16]. Using a variance component methodology, Cripe et al. found that, using their mathematical model, the heritability of BAV was 89% suggesting that BAV is almost entirely genetically determined and that mutations in diverse genes with divergent inheritance pattern may be responsible for BAV formation in different families [19]. In conclusion, epidemiologic studies suggest that BAV is heritable and follows an autosomal dominant mode of transmission with reduced penetrance and variable expressivity.

While the heritability of BAV is now well established, genes linked to the defect remain largely unknown. A family-based linkage analysis using microsatellite markers revealed linkage to three loci on chromosome 18q, 5q, and 13q, but the precise gene(s) within these regions were not defined [20]. To date, only mutations in *NOTCH1*, a gene that maps to 9q34-35, have been associated with BAV in a small number of families (Table 1). Initially, a nonsense and a frameshift mutation in two family members with BAV and valve calcification were identified in a large family with 11 cases of congenital heart disease, four of which required aortic valve replacement [21]. Shortly thereafter, new undescribed missense mutations were identified in patients with BAV and/or aortic aneurysms in two independent studies [22, 23]. These observations provided strong evidence that *NOTCH1* haploinsufficiency can cause aortic valve disease.

Mutations in transcription factor GATA6 have been associated with congenital heart defects, including tetralogy of Fallot, persistent truncus arteriosus, and atrial septal defects [24–26]. *GATA6* is involved in OFT morphogenesis and its deletion from neural crest cells in mice causes perinatal lethality due to a spectrum of aortic arch patterning and cardiac OFT septation defects [27]. Recently, Lin et al. discovered that one of the parents of a patient with ASD had a BAV suggesting a possible relationship between GATA6 and BAV (Table 1). Interestingly, GATA6 maps to chromosome 18q, which is linked to BAV, but no mutations in the *GATA6* gene have been reported yet in individuals with BAV [20]. Of note, examination of the aortic valve of *Gata6* heterozygote mice revealed partial penetrance of BAVs (our unpublished data) supporting a role for GATA6 as a candidate BAV-causing gene.

Human genetics have also revealed that perturbations in the expression of ECM components (elastin, collagen, and proteoglycans) can lead to cardiac defects (Table 1). For example, mutations in* FBN1* (an extracellular glycoprotein of the ECM that acts to maintain tissue elasticity of the valve leaflets and aortic wall by linking smooth muscle cells to adjacent elastin fibrils) have been observed in patients with Marfan syndrome (MFS), a genetic disorder of the connective tissue characterized by mitral valve prolapse, BAV, and/or aortic dissection and aneurysm [28–30]. Moreover, reduced expression of* FBN1* in the aorta of patients with BAVs was reported [31]. Of note, targeted deletion of* Fbn1* in mice phenocopies the defects observed in Marfan syndrome indicative of a causal relationship between *FBN1* mutations and valve malformation [32, 33]. These mice have upregulated TGF-*β* signalling, mitral valve prolapse and die shortly after birth due to aortic dissection caused by weakening of the aortic wall [34]. Interestingly, mutations in the TGF-*β* receptors, *TGFBR1* and *TGFBR2*, have been associated with Marfan and Loeys-Dietz syndromes [35, 36]. Recently, a missense mutation in *TGF*β*R2* identical to the one found in MFS patients who tested negative for mutation in FBN1 was found in a patient with BAV and aortic aneurysm [37] but an earlier study found no mutation in either TGFBR1 or TGFBR2 in patients with isolated BAV.

Aortic dilation, dissection, and/or aortic aneurysm are the most common vascular complications in patients with BAV. Linkage analysis of 7 family members with aortic aneurysms and dissection, of whom three had BAVs identified *ACTA2* which encodes smooth muscle *α*-actin [38]. Analysis of aortic tissue from these patients showed increased proteoglycans accumulation, fragmentation, loss of elastic fibers, and decreased numbers of smooth muscle cells, consistent with aortic wall degeneration. However, whether *ACTA2* mutations cause BAV remains uncertain.

Other gene mutations have been linked to syndromic disease that include aortic valve abnormalities. For example, homozygous truncating mutations in *HOXA1* have been associated to Bosley-Salih-Alorainy syndrome and Athabascan Brainstem Dysgenesis syndrome [39]. Interestingly, severe cardiovascular malformations, including interrupted aortic arch type B, aberrant subclavian artery, ventricular septal defect, tetralogy of Fallot, and BAV, are observed in these syndromes. Targeted inactivation of *Hoxa1* in mice was shown to recapitulate these defects [40]. Among cardiac malformations, BAVs were obtained with a prevalence of 24%. It remains to be seen whether mutations in *HOXA1* will be discovered in BAV patients.

Similarly, heterozygous missense mutations in the *KCNJ2* potassium channel have been linked to Andersen syndrome (Table 1) [41]. This rare disorder is characterized by prolongation of the QT interval with ventricular arrhythmias, periodic paralysis, dysmorphic facies, cleft palate, and scoliosis. Additional features seen in the pedigree were cardiovascular malformations, including BAV, BAV with coarctation of the aorta, or pulmonary stenosis, which had never been associated with this disease before. Whether this or another as yet unidentified mutation causes BAV is unclear and the link between defective potassium current and abnormal aortic valve formation has not been investigated.

In summary, human genetics support the involvement of multiple causative and modifier genes in BAV inheritance. The majority of these genes remain to be identified.

## 3. Insights from Animal Models

Over the past decade, analysis of aortic valves of genetically engineered mice have provided important insight into valve development and identified potential candidate genes that could underlie BAV formation (Table 2). One of the first mouse models of BAV was reported in *Nos3* null mice who had a broad spectrum of CHD including BAV all from the fusion of the right and noncoronary leaflet (R-N) [9, 42]. *Nos3* is expressed in endocardial cells of the heart and is shear-stress-dependent [43]. Since formation of the endocardial cushions depends on the EMT, a shear-stress dependent process, *Nos3* deficiency might alter endocardial cell migration during EMT, causing abnormal development of the valve cushion. Consistent with this, *NOS3* expression was found to be significantly lower in patients with BAV [44], providing further support for the relevance of NOS3 and possibly its regulators in BAV formation.

Members of the GATA family of transcription factors such as the endothelially expressed GATA2 are transcription activators of *NOS3* [45]. Another member, GATA5, has been shown to be restricted to endocardial cells and the endocardial cushions of the AV canal and OFT [46]. Recently, we generated a *Gata5* null mouse model and found partial penetrance of BAVs with a prevalence of 26% [47]. To gain more insight into the role of Gata5 in endocardial development including the cell type contributing to these defects, we generated a conditional mouse model lacking *Gata5* only in endothelial cells (*eGata5^−/−^*). The *eGata5* mutant mice had a similar prevalence of BAVs as the *Gata5* null mice suggesting a cell autonomous function for Gata5 in endocardial cushion development. Remarkably, in both mouse models, the BAV was due to fusion of the right and noncoronary leaflets (R-N BAV), identical to what was seen in the *Nos3* null mice. Reduced expression of *Nos3* was observed in the endocardial cushions of the OFT in the *Gata5* null mice. Together these observations point to exquisite NOS3 dosage sensitivity in aortic leaflet formation. *In vitro* studies demonstrated that Gata5 is a potent activator of the *Nos3* promoter suggesting that *NOS3* may be the downstream effector of GATA5 in endocardial cushion cells. Our studies also revealed that the Notch pathway is significantly downregulated in *Gata5* null and *eGata5^−/−^* mice. Of note, decreased expression of *Jag1* the Notch ligand was observed in *Gata5* null and *eGata5^−/−^* mice with a concomitant upregulation of Rbpj-*κ*, the Notch pathway repressor. Downregulation of the Notch pathway in embryonic hearts was confirmed by decreased immunostaining for the Notch1 intracellular domain (NCID). Together, these observations suggest that reduced Notch1 activation and subsequent Notch signaling in the outflow tract could contribute to abnormal endocardial cushion formation and fusion of the aortic valve leaflets. The Notch pathway plays major roles in multiple developmental processes, including cardiovascular development. Among others, it has been shown to be critical for EMT, a key process for valve formation. In mammals, the Notch family consists of 4 type I transmembrane receptors (Notch1 to 4) and 5 type I transmembrane ligands, Jagged1, Jagged2, Delta-like (Dll) 1, Dll3, and Dll4 [48]. Upon ligand binding, a protease complex containing gamma secretase cleaves the intracellular domain of Notch, which enters the nucleus and regulates gene expression through binding to the transcription factor RBP-J*κ*. It has been observed that Notch1 plays key roles during valve development and EMT, consistent with its expression pattern in the endocardium and OFT cushion mesenchyme. Of note, *Notch1* null mice die early due to severe cardiac defects, including defective EMT [49]. In the last couple of years, different groups have tried to shed light on the molecular mechanism of aortic valve calcification. Nigam and Srivastava reported that inhibition of *Bmp2* blocked calcification of murine aortic valves *in vivo* and *in vitro*, suggesting that Notch1 represses Bmp2 within the aortic valve [50]. In addition, Acharya et al. discovered that inhibition of Notch1 in an *in vitro* model of aortic valve calcification was prevented by the addition of Sox9, indicating that Notch1 regulates aortic valve calcification through a Sox-9-dependent pathway [51]. In conclusion, all of these findings demonstrate that *NOTCH1* haploinsufficiency plays a key role in valvulogenesis as well as the maintenance of normal valve function in the adult heart. Expression of the three cardiac GATA factors partially overlaps during cardiac development. Our studies revealed that *Gata4^+/−^Gata5^+/−^* and *Gata5^+/−^Gata6^+/−^* die embryonically or perinataly due to profound cardiac defects including double outlet right ventricles and ventricular septal defects [52]. Interestingly, nearly half (3/7) *Gata4^+/−^Gata5^+/−^* mice had very high pressure gradient through the aortic valve and examination of the aortic valve of these mice revealed the presence of BAVs. Similarly, we observed BAV in 25% (1/4) *Gata5^+/−^Gata6^+/−^* embryos at E18.5 (unpublished data). These observations are indicative of dosage sensitivity for cardiac GATA factors in aortic valve formation. They also suggest that CHD-causing genes might interact to influence CHD penetrance and expressivity including BAV. These results have important implications for human genetic studies.

BAVs have been reported in a small proportion (11%) of mice haploinsufficient for *Nkx2.5 *[53], a gene that has been associated with several human CHDs (Table 2). *NKX2.5* maps to chromosome 5q34, which has been linked to BAVs, and although one study did not find any polymorphisms in the coding region of *Nkx2.5 *in BAV patients [54], it still remains to be determined whether mutations in the *NKX2.5* gene will be discovered in future studies of human BAV. Nkx2.5 is a critical regulator of cardiac morphogenesis and was shown to modulate ECM of the aorta through regulation of collagen type I [55]. Interestingly, mutations in collagen type I have been linked to Ehlers-Danlos syndrome, which is characterized by skin and bone abnormalities as well as mitral and aortic valve dysfunction [56].

In conclusion, while the molecular basis of BAV is incompletely understood, at least 2 pathways seem critical for normal tricuspid formation, namely, Notch and Nos3. This information, together with the availability of mouse models of BAV, represents an important step in understanding the pathogenesis of BAV and for studies aimed at the prevention of BAV associated cardiovascular events.

## 4. Is BAV Related to Other Congenital Heart Defects?

 In general most inherited congenital heart defects (CHDs) show variable expressivity and penetrance including many autosomal dominant syndromes. As stated earlier, BAVs have been identified in family members whose parents or siblings had other CHDs. The availability of the Gata5 null mice which do not have other structural heart defects besides BAV offers the unique possibility to genetically test the link between BAV and other heart defects. This is easily achieved by crossing with specific mouse strains. The first set of experiments aimed to explore the consequences of combinatorial heterozygosity of Gata5 and the 2 other cardiac GATA factors, GATA4 and GATA6, both of which have been linked to human CHD including septal and valve defects [24, 26, 57–59].

 The resulting double hets offsprings had multiple CHDs and reduced survival suggesting that genes linked to BAVs contribute to other cardiac defects. This information is important for understanding the incidence of BAVs in conjunction with other CHDs in families and for future human genetic studies.

## 5. Where Do We Go from Here?

BAVs as well as other CHDs are complex multifactorial and multigenetic diseases with variable expressivity and penetrance. Thus, not all family members with a similar gene mutation will have the same heart disease and the same mutation can cause different CHDs in different individuals. These observations are consistent with the presence of modifying factors including genetic and environmental ones that influence the phenotype [60]. Consistent with this, influences of the genetic background on the phenotype is now well documented in experimental animal models and in humans [59, 61, 62]. Thus, analysis of upstream regulators, interacting proteins as well as downstream targets of genes known to be linked to BAV will likely identify new CHD-causing or modifier genes.

Moreover, understanding the progression of the disease starting in childhood would help to slow down disease progression and improve the timing of intervention of each patient. In addition, BAV-linked genes have also been shown to be involved in other adaptive responses of the heart. For example, *Notch1*-deficient mice subjected to pressure overload develop increased hypertrophy, fibrosis and mortality rate is increased, suggesting that Notch1 is important for survival [63]. Similarly, *Gata5* null mice subjected to pressure overload develop more cardiac conduction defects, have increased fibrosis, cardiac hypertrophy, and heart failure (unpublished data).

Such studies combined with our ability to delete genes in specific cells and at specific developmental states in animal models will undoubtedly unravel numerous candidate CHD-causing genes that can be directly tested in human genetic studies. Conversely *loci* identified through linkage analysis in human cohorts can be tested in animal models to confirm (or not) their causative link to disease.

Additionally, the availability of animal models of BAV and other valve disease will allow direct testing and identification of genetic modifiers as well as the molecular basis of gene-environment interaction in BAV formation.

Lastly, BAV is a risk factor for early onset of serious cardiovascular complications but the molecular basis and tools (such as biomarkers) to monitor disease progression remain largely undefined.

The existing (and future) animal models of disease will hugely facilitate such important analysis by allowing manipulations of diets, of cardiovascular parameters such as volume and pressure overload, and of other aging-related alterations. The results of such studies will undoubtedly be translated into better diagnosis followup and prevention of premature complications in individuals with family history of valve disease.

## Figures and Tables

**Table 1:** Human gene mutation and phenotype associated with bicuspid aortic valve formation.

Gene affected	Human syndrome	Human cardiac phenotype	Mutation and/or expression in BAV patients	Mouse valve phenotype	Reference
NOTCH1		BAV, calcification, BAV with aortic aneurysm	R1108X, H1505del, T596M, P179H, A1343V, P1390T	Thick valve leaflets, calcification	[21–23]
GATA6		ASD, TOF	Mother of the ASD patient had a BAV	No valve phenotype reported	[26]
FBN1	Marfan syndrome	Mitral valve prolapse, Aortic aneurysm	Reduced expression of FBN1	Thick and long valve leaflets, mitral valve prolapse	[28–31]
TGFBR2	Loeys-Dielz syndrome, Marfan syndrome	Mitral valve prolapse, aortic aneurysm, BAV	V387M	Defective remodelling of the AV cushion, lethality at E11.5 precluding analysis of valves	[36]
ACTA2		Thoracic aortic aneurysm and dissection	Three family members had a BAV	No valve phenotype	[38]
HOXA1	Bosley-Salih-Alorainy syndrome, athabascan brainstem dysgenesis syndrome	Interrupted aortic arch type B, aberrant subclavian artery, VSD, TOF, BAV	Not reported	BAV	[39]
KCNJ2	Andersen syndrome	BAV, BAV with coarctation of the aorta, pulmonary stenosis	R67W	Not reported	[41]

ASD: atrial septal defect; BAV: bicuspid aortic valve; OFT: outflow tract; TOF: tetralogy of Fallot; VSD: ventricular septal defect.

**Table 2:** Mouse models of bicuspid aortic valve formation.

Gene affected	Human cardiac phenotype	Mouse model	Mouse valve phenotype	Cardiovascular expression	Reference
Hoxa1	IAAB, ASC, VSD, BAV, TOF	*Hoxa1^−/−^*	BAV	Neural crest cells, OFT endocardium and myocardium, Secondary heart field	[40]
NOS3	Not reported	*Nos3^−/−^*	BAV	Endocardium, myocardium	[42]
Gata5	Not reported	*Gata*5*^−/−^* and *Tie*2*^Cre^*; *Gata*5^F/F^	BAV	Endocardium, AV and OFT endocardial cushions, subset of endocardial cells, epicardium	[47]
Nkx2.5	ASD, AV block, VSD, TOF, HCM	*Nkx2.*5^GFP^ *het*, *Nkx2.*5^HDneo^ *het *	BAV, aortic aneurysm	Myocardium	[53]

ASC: aberrant subclavian artery; ASD: atrial septal defect; AV: atrioventricular; BAV: bicuspid aortic valve; HCM: hypertrophic cardiomyopathy; IAAB: interrupted aortic arch type B; VSD: ventricular septal defect; TOF: tetralogy of Fallot.
